# Antiarrhythmic Effects of Vernakalant in Human-Induced Pluripotent Stem Cell-Derived Cardiomyocytes from a Patient with Short QT Syndrome Type 1

**DOI:** 10.3390/jcdd9040112

**Published:** 2022-04-09

**Authors:** Qiang Xu, Xuemei Huang, Zenghui Meng, Yingrui Li, Rujia Zhong, Xin Li, Lukas Cyganek, Ibrahim El-Battrawy, Ibrahim Akin, Xiaobo Zhou, Huan Lan

**Affiliations:** 1 School of Basic Medical Science, Southwest Medical University, Luzhou 646000, China; xlunasea@swmu.edu.cn; 2 First Department of Medicine, Faculty of Medicine, University Medical Centre Mannheim (UMM), University of Heidelberg, 68167 Mannheim, Germany; zenghui.meng@medma.uni-heidelberg.de (Z.M.); yingrui.li@medma.uni-heidelberg.de (Y.L.); rujia.zhong@medma.uni-heidelberg.de (R.Z.); xin.li@medma.uni-heidelberg.de (X.L.); ibrahim.elbattrawy2006@gmail.com (I.E.-B.); ibrahim.akin@umm.de (I.A.); 3 Key Laboratory of Medical Electrophysiology, Ministry of Education & Medical Electrophysiological Key Laboratory of Sichuan Province, Collaborative Innovation Center for Prevention of Cardiovascular Diseases, Institute of Cardiovascular Research, Southwest Medical University, Luzhou 646000, China; 20194099120016@stu.swmu.edu.cn; 4 Stem Cell Unit, Clinic for Cardiology and Pneumology, University Medical Center Göttingen, 37075 Göttingen, Germany; lukas.cyganek@gwdg.de; 5 DZHK (German Center for Cardiovascular Research), Partner Site, 37073 Göttingen, Germany; 6 DZHK (German Center for Cardiovascular Research), Partner Site, 68229 Heidelberg-Mannheim, Germany

**Keywords:** short QT syndrome, arrhythmias, antiarrhythmic drugs, vernakalant, human-induced pluripotent stem cell-derived cardiomyocytes

## Abstract

(1) Background: Short QT syndrome (SQTS) may result in sudden cardiac death. So far, no drugs, except quinidine, have been demonstrated to be effective in some patients with SQTS type 1 (SQTS1). This study was designed to examine the potential effectiveness of vernakalant for treating SQTS1 patients, using human-induced pluripotent stem cell-derived cardiomyocytes (hiPSC-CMs) from a patient with SQTS1. (2) Methods: Patch clamp and calcium imaging techniques were used to examine the drug effects. (3) Results: Vernakalant prolonged the action potential duration (APD) in hiPSC-CMs from a SQTS1-patient (SQTS1-hiPSC-CMs). In spontaneously beating SQTS1-hiPSC-CMs, vernakalant reduced the arrhythmia-like events induced by carbachol plus epinephrine. Vernakalant failed to suppress the hERG channel currents but reduced the outward small-conductance calcium-activated potassium channel current. In addition, it enhanced Na/Ca exchanger currents and late sodium currents, in agreement with its APD-prolonging effect. (4) Conclusions: The results demonstrated that vernakalant can prolong APD and reduce arrhythmia-like events in SQTS1-hiPSC-CMs and may be a candidate drug for treating arrhythmias in SQTS1-patients.

## 1. Introduction

Short QT syndrome (SQTS) is a rare, inheritable heart disease, characterized by a shortened corrected QT interval (QTc) on the electrocardiogram (ECG). It was first described in 2000 and the first gene mutation of N588K in the KCNH2 gene was detected in 2004 [1,2]. Thereafter, more and more SQTS patients with different gene mutations have been reported and different types of SQTS have been described [3]. SQTS types 1–3 are associated with a gain in function of potassium channels, caused by mutations in the KCNH2 (SQTS1), KCNQ1 (SQTS2) and KCNJ2 (SQTS3) gene. SQTS types 4–6 are associated with a loss of function in calcium channels, caused by mutations in the CACNA1C (SQTS4), CACNB2 (SQTS5) and CACNA2D1 (SQTS6) genes. Recently, a mutation in the cardiac Cl/HCO_3_ exchanger AE3 was detected in two SQTS families [4]. Because of the low prevalence and rare cases, the diagnostic and treatment approaches for SQTS are still challenging. Although an implantable cardioverter defibrillator (ICD) can be used in survivors of SCD [5], ICD has side effects and cannot be used for every patient [6,7]. Therefore, pharmacotherapy is recommended as an adjunctive treatment for SQTS.

Until now, only a small number of drugs have been reported to be examined in a small number of patients with SQTS1 [8,9], among which only quinidine has shown beneficial effects in the treatment [9,10]. Unfortunately, quinidine has been removed from the market in several countries and often has intolerable side-effects, including the ventricular pro-arrhythmic effect of repetitive and paroxysmal torsade de pointes (TdP) tachycardias, with recurrent syncope and even sudden cardiac death, gastrointestinal side effects and electrolyte imbalances, etc. More drugs are needed for SQTS treatment. 

SQTS1 resulted from a gain-of-function in the hERG channel. hERG channel blockers should prolong the QT interval and, hence, suppress arrhythmias in SQTS1 patients. Surprisingly, clinical data demonstrated that some hERG channel blockers, including sotalol and ibutilide, failed to prolong the QT interval in SQTS1 patients with hERG mutations [11]. The reason for the ineffectiveness of hERG channel blockers in SQTS1 patients carrying the mutation (hERG-N588K) is that the mutation impairs the inactivation of the channel and renders the channel resistant to blockers, which have high affinity to the inactivated state of hERG channels. Quinidine has the same affinity to the open and inactivated states of hERG channels. Hence, the inactivation defect caused by the mutation only partially reduced the affinity of quinidine and quinidine is effective in treating SQTS1. Searching for more drugs, which can be as effective as quinidine, is required for the treatment of SQTS. Given that a mutation in KCNH2 (or other channels) may change the sensitivity of channels to drugs [12], effects of candidate drugs for SQTS should be tested in cardiomyocytes with the mutation. An optimal model of SQTS with a certain gene mutation warrants studies searching for effective drugs for treating SQTS, at least the SQTS with the same pathogenic gene mutation.

In our recent studies, we have successfully generated human-induced pluripotent stem cell-derived cardiomyocytes (hiPSC-CMs) from a patient with SQTS type 1. The hiPSC-CMs from the patient (SQTS1-hiPSC-CMs) recapitulated the main features of the disease (APD shortening and arrhythmias) and responded to drugs (e.g., quinidine and sotalol), in the same manner (quinidine but not sotalol was effective) as the heart cells in SQTS1 patients do [11,13,14]. This indicates that the SQTS1-hiPSC-CMs can be used as a cellular model for either mechanistic or therapeutic studies on SQTS1. 

Vernakalant, an antiarrhythmic drug developed for selective treatment of atrial fibrillation, is a multi-channel blocker (sodium and potassium channel blocker). It has been tested in rabbits with acquired SQTS [15]. In that study, SQTS was induced by an ATP-sensitive K channel opener (pinacidil). Vernakalant significantly prolonged the APD and QT interval and reduced the occurrence of ventricular fibrillation induced by pinacidil. To date, vernakalant has not been tested in SQTS patients or cardiomyocytes from SQTS patients. Whether vernakalant is effective in SQTS patients is so far unknown. This study was designed to test the effects of vernakalant on APD and arrhythmia-like events in our established cellular model of SQTS1-hiPSC-CMs.

## 2. Methods

### 2.1. Ethics Statement

A skin biopsy from an SQTS1 patient was obtained with written informed consent. The study was approved by the Ethics Committee of the Medical Faculty Mannheim, University of Heidelberg (approval numbers: 2018-565N-MA) and by the Ethics Committee of University Medical Center Göttingen (approval number: 10/9/15). The study was carried out in accordance with the approved guidelines and conducted in accordance with the Helsinki Declaration of 1975 revised in 2013 [16].

### 2.2. Clinical Data

A male patient with familial SQTS1 was recruited for the study. He carries a missense mutation (c.1764C > G) resulting in substitution of an amino acid at the position of 588 from asparagine to lysine (N588K) in hERG channel. The clinical data of the patient were provided in our recent publication [14]. In brief, the patient was a 29-year-old male patient when the skin biopsy was performed in 2016. The SQTS was diagnosed in 2003. There is a strong family history of SCD in his family. Three members of the family including this patient presented a short QT interval (270–300 ms) corrected with Bazett’s formula (QTc) at ECG. The extensive clinical and instrumental evaluation confirmed the familial SQTS.

### 2.3. Generation of Human iPS Cells

The methods for the generation of iPS cells from the patient were described in our previous study [14]. Briefly, human iPS cells (hiPSCs) were generated from primary human fibroblasts derived from a skin biopsy. The hiPSC line was generated in feeder-free culture conditions using the integration-free CytoTune-iPS 2.0 Sendai Reprogramming Kit (Thermo Fisher Scientific, #A16517, Frederick, MD, USA) with the reprogramming factors OCT4, KLF4, SOX2, c-MYC according to manufacturer’s instructions with modifications. The generated hiPSCs were characterized for their pluripotency and their in vitro differentiation potential [14]. 

### 2.4. Generation of hiPSC-CMs 

The hiPSCs were cultured in Matrigel-coated flask and differentiated into hiPSC-CMs as described with some modifications [17]. In our lab, the differentiation of hiPS cells into cardiomyocytes (hiPSC-CMs) was regularly carried out every 2 to 3 weeks. The hiPSC-CMs from different differentiations were used for studies and the data were combined. At 40 to 60 days of differentiation, cardiomyocytes were dissociated from 24-well plates and plated on Matrigel-coated 3.5 cm petri dishes for patch-clamp and calcium transient measurements.

### 2.5. Patch-Clamp 

Standard patch-clamp whole-cell recording techniques were used to measure the action potential (AP) and channel currents at room temperature (±26 °C). Patch electrodes were pulled from borosilicate glass capillaries (MTW 150F; world Precision Instruments, Inc., Sarasota, FL) using a DMZ-Universal Puller (Zeitz-Instrumente Vertriebs GmbH, Martinsried, Germany) and filled with pre-filtered pipette solution (see below). Pipette resistance ranged from 1–2 MΩ and 3–4 MΩ for current and AP measurements, respectively. Signals were acquired at 10 kHz and filtered at 2 kHz with the Axon 200B amplifier and Digidata 1440A digitizer hardware as well as pClamp10.2 software (Molecular Devices, Sunnyvale, CA, USA). APs were recorded in current clamp mode. For recording APs, brief current pulses (2 ms, 1 nA) were applied with different frequencies to trigger APs. A hyperpolarizing current of 10 pA was injected during APs’ recording to abort the automaticity of hiPSC-CMs.

The bath solution (PSS) for AP measurements contained (mM): 127 NaCl, 5.9 KCl, 2.4 CaCl_2_, 1.2 MgCl_2_, 11 glucose, 10 HEPES, pH 7.4 (NaOH). The pipette solution contained (mM): 10 HEPES, 126 KCl, 6 NaCl, 1.2 MgCl_2_, 5 EGTA, 11 glucose and 1 MgATP, pH 7.2 (KOH). 

The bath solution for L-type (I_Ca-L_) calcium channel current recordings contained (mM): 140 TEA-Cl, 5 CaCl_2_, 1 MgCl_2_, 0.001 E-4031, 10 HEPES, 10 glucose, 0.02 TTX, 3 4-AP, pH 7.4 (CsOH). Microelectrodes were filled with (mM): 10 NaCl, 135 CsCl, 2 CaCl_2_, 3 MgATP, 5 EGTA, 10 HEPES, pH7.2 (CsOH). 

The bath solution for peak sodium current (peak I_Na_) recordings contained (mM): 20 NaCl, 130 CsCl_2_, 1.8 CaCl_2_, 1 MgCl_2_, 10 glucose, 10 HEPES, 0.003 nifedipine, pH 7.4 (NaOH). The bath solution for late sodium current (late I_Na_) recordings contained (mM): 135 NaCl, 20 CsCl_2_, 1.8 CaCl_2_, 1 MgCl_2_, 10 glucose, 10 HEPES, 0.003 nifedipine, pH 7.4 (NaOH). The pipette solution contained (mM): 10 HEPES, 135 CsCl_2_, 2 CaCl_2_, 5 EGTA, 3 MgATP, pH 7.2 (CsOH).

The bath solution for Na/Ca exchanger current (I_NCX_) measurements contained (mM): 135 NaCl, 10 CsCl, 2 CaCl_2_, 1 MgCl_2_, 10 HEPES, 10 glucose, 0.01 nifedipine, 0.1 niflumic acid, 0.05 lidocaine, 0.02 dihydroouabain, pH 7.4 (CsOH). Microelectrodes were filled with (mM): 10 NaOH, 150 CsOH, 2 CaCl_2_, 1 MgCl_2_, 75 aspartic acid, 5 EGTA, pH7.2 (CsOH). I_NCX_ was defined as NiCl_2_-sensitive current.

The bath solution for transient outward current (I_to_) recordings contained (mM): 127 NaCl, 5.9 KCl, 2.4 CaCl_2_, 1.2 MgCl_2_, 11 glucose, 10 HEPES, 0.01 nifedipine, 0.003 E-4031, 0.01 TTX, pH 7.4 (NaOH). The pipette solution contained (mM): 10 HEPES, 126 KCl, 6 NaCl, 1.2 MgCl_2_, 5 EGTA, 11 glucose and 1 MgATP, pH 7.2 (KOH).

The bath solution for K^+^ channel current measurements contained (mM): 127 NaCl, 5.9 KCl, 2.4 CaCl_2_, 1.2 MgCl_2_, 11 glucose, 10 HEPES, pH 7.4 (NaOH). For slowly delayed rectifier (I_Ks_) measurements, 10 µM nifedipine, 3 mM 4-AP and 10 µM TTX were added. The pipette solution contained (mM): 10 HEPES, 126 KCl, 6 NaCl, 1.2 MgCl_2_, 5 EGTA, 11 glucose and 1 MgATP, pH 7.4 (KOH). For measuring small conductance calcium-activated potassium channel currents (I_SK_), appropriate CaCl_2_ was added to get the free-Ca^2+^ concentration of 0.5 µM according to the calculation by the software MAXCHELATOR. For measuring ATP-sensitive K^+^ channel currents (I_KATP_), the ATP-free pipette solution was used. I_Ks_ was defined as 3R4S-chromanol 293B-sensitive, I_KATP_ as glibenclamide-sensitive and I_SK_ as apamin-sensitive currents.

To improve I_Kr_ measurements, the Cs^+^ currents conducted by KCNH2 (I_Kr_) channels were measured. External solution for Cs^+^ currents (mM): 140 CsCl_2_, 2 MgCl_2_, 10 HEPES, 10 Glucose, pH = 7.4 (CsOH). Pipette solution: 140 CsCl_2_, 2 MgCl_2_, 10 HEPES, 10 EGTA, pH = 7.2 (CsOH).

### 2.6. Measurement of Intracellular Calcium Transients 

To measure the intracellular Ca^2+^ transients, cells were loaded with the fluorescent Ca^2+^-indicator Fluo-3 AM. First, 1.5 mL PSS (see below) was added into a petri dish with hiPSC-CMs cultured for 2 to 4 days. Then, 50 µg of the membrane permeable acetoxymethyl ester derivative of Fluo-3 AM was dissolved in 44 µL of the Pluronic F-127 stock solution (20% *w*/*v* in DMSO) to get a 1 mM Fluo-3 AM stock solution, which can be stored at −20 °C for a maximum of 1 week. Next, 15 µL of the Fluo-3 AM stock solution was added into 1.5 mL PSS resulting in a final concentration of 10 µM Fluo-3 and the dish was agitated carefully. The cells were incubated at room temperature for 10 min in an optically opaque box to protect from light. Thereafter, the PSS was carefully pipetted out and discarded and the cells were washed with PSS 4–5 times. Finally, the cells in PSS were kept at room temperature for about 30 min for de-esterification before measurements. After de-esterification the fluorescence of the cells was measured by using Cairn Optoscan calcium imaging system (Cairn Research, UK). Fluorescence is excited by 488 nm and emitted at 520 nm. 

### 2.7. Drugs

Vernakalant was from Sigma-Aldrich (Taufkirchen, Germany). The drug was applied to a cell by a perfusion pipette, from low to high concentrations. The used concentrations were determined according to previous or our preliminary studies in hiPSC-CMs. E-4031, chromanol 293B, nifedipine, NiCl_2_, glibenclamide, niflunic acid, lidocaine and dihydroouabain were from Sigma-Aldrich, 4-AP from RBI, apamin from Alomone Labs, tetrodotoxin (TTX) from Carl Roth (Karlsruhe, Germany). E-4031, NiCl_2_, TTX, 4-AP, apamin, niflumic acid and dihydrooubain were dissolved in H_2_O. Nifedipine, and chromanol 293B were dissolved in DMSO, lidocaine in ethanol. Stock solutions were kept at −20 °C. 

### 2.8. Statistical Analysis

Data are shown as mean ± SEM and were analyzed using InStat© (GraphPad, San Diego, CA, USA) and SigmaPlot 11.0 (Systat GmbH, Düsseldorf, Germany). For parametric data of more than two groups multiple comparisons with one-way ANOVA and Holm–Sidak post-test were performed. Paired t-test was used for comparisons of data before and after application of a drug. *p* < 0.05 (two-tailed) was considered significant.

## 3. Results

### 3.1. Effects of Vernakalant on Action Potentials (APs) 

**Figure 1:**
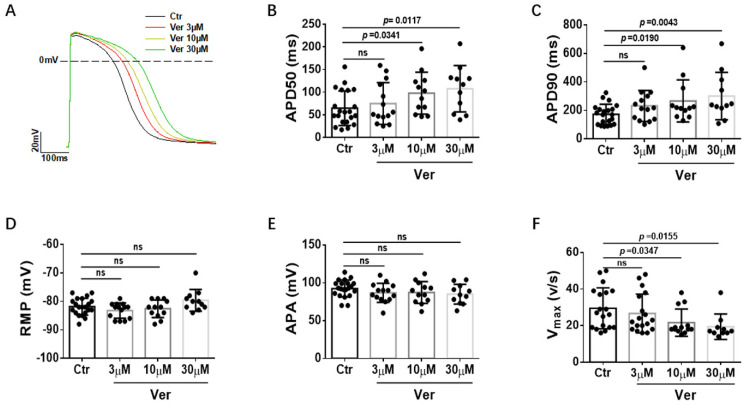
Effects of vernakalant on action potentials in SQTS1-hiPSC-CMs. (**A**) Representative action potential traces in absence (Ctr) and presence of 3 µM, 10 µM and 30 µM vernakalant. (**B**) Averaged values of action potential duration at 50% repolarization (APD50). (**C**) Averaged values of action potential duration at 90% repolarization (APD90). (**D**) Averaged values of resting membrane potential (RMP). (**E**) Averaged values of action potential amplitude (APA). (**F**) Averaged values of maximal depolarization velocity (V_max_). All the action potentials were recorded at 1 Hz. Data are shown as mean ± SEM from 21 cells. The statistical significance was examined by One Way Repeated Measures ANOVA followed by Holm–Sidak method.

Vernakalant at different concentrations (3 µM, 10 µM and 30 µM) was applied to SQTS1-hiPSC-CMs to check its effects on APs triggered by stimulations at 1 Hz. The AP parameters, including action potential amplitude (APA), the maximal upstroke velocity (V_max_) and action potential durations at 50% and 90% repolarization (APD50 and APD90), were analyzed. Vernakalant at the concentration of 10 µM and 30 µM significantly prolonged APD. At 10 µM, APD50 was prolonged from 64.4 ± 8.4 ms to 97.6 ± 13.3 ms and APD90 from 172.7 ± 15.4 ms to 266.4 ± 42.6 ms, whereas at 30 µM, APD50 was prolonged to 107.4 ± 15.4 ms and APD90 to 301.2 ± 50.1 ms (Figure 1A–C). Vernakalant at all the tested concentrations failed to affect resting membrane potential (RMP) and APA (Figure 1D,E), but reduced V_max_ in a concentration-dependent manner (Figure 1F), consistent with its Na channel-blocking effect. In hiPSC−CMs from the healthy donor, vernakalant showed similar effects on AP parameters (Figure S1).

**Figure 2:**
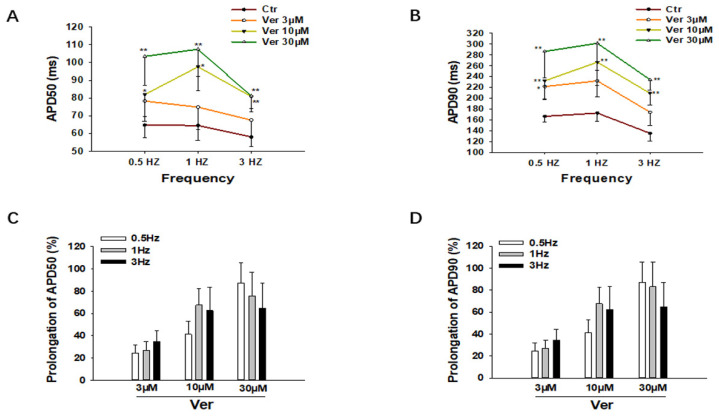
Vernakalant prolonged APD at different frequencies. (**A**,**B**) Averaged values of APD50 and APD90 at 0.5 Hz, 1 Hz, and 3 Hz in absence (Ctr) and presence of 3 µM, 10 µM and 30 µM vernakalant from 21 cells. (**C**,**D**) Percent prolongation of APD50 and APD90 by vernakalant at 0.5 Hz, 1 Hz, and 3 Hz. The values were calculated from the data in A and B. Data are shown as mean ± SEM, the statistical significance was examined by One Way Repeated Measures ANOVA followed by Holm–Sidak method. * *p* < 0.05, ** *p* < 0.01.

To check the effects of vernakalant at different frequencies, the same measurements were repeated in cells paced by stimulations at 0.5 Hz, 1 Hz and 3 Hz. As expected, vernakalant prolonged APDs at all the three frequencies, without clear frequency dependence (Figure 2). 

### 3.2. Vernakalant Reduced Arrhythmia-Like Events in SQTS1-hiPSC-CMs 

**Figure 3:**
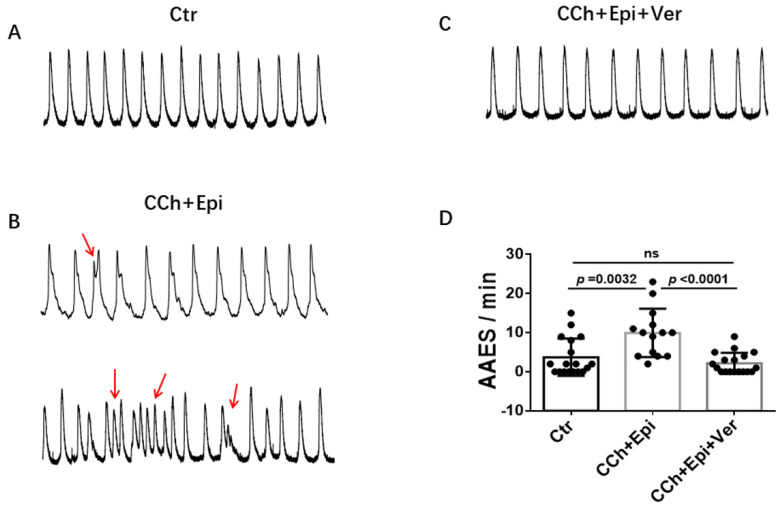
Vernakalant reduced arrhythmia-associated events. Calcium transients were measured in spontaneously beating cells. Then carbachol (10 µM) plus epinephrine (10 µM) was applied to cells to trigger arrhythmia-associated events (AAEs). In cells showing AAEs, vernakalant (Ver, 10 µM) was applied to the cell in presence of carbachol and epinephrine. (**A**) Representative traces of calcium transients in a cell before challenging (Ctr). (**B**) Representative traces of calcium transients in the cell challenged by carbachol plus epinephrine (CCh+Epi). (**C**) Representative traces of calcium transients in the cell in the presence of carbachol plus epinephrine and vernakalant (CCh+Epi+Ver). (**D**) Averaged values of AAEs per minute. CCh+Epi slowed the beating but led to small and irregularly triggered beating. The AAEs were defined as transients that are larger than 10% but smaller than 80% of the normal regular transients. Data are shown as mean ± SEM from 18 cells. *p* values were determined by One Way ANOVA analysis followed by Holm-Sidak method.

Due to the APD-prolonging effect of vernakalant, its effects on arrhythmias were further assessed in cells showing arrhythmia-like events triggered by carbachol (CCh, 10 µM) plus epinephrine (Epi, 10µM). In spontaneously beating cells, spontaneous calcium transients were measured to monitor arrhythmia-like events. CCh+Epi elevated the episodes of arrhythmia-like events, such as DAD- (delayed after depolarization) like and EAD- (early after depolarization) like events. Vernakalant reduced the arrhythmia-like events induced by CCh+Epi (Figure 3). 

### 3.3. Effects of Vernakalant on Ion Channel Currents in hiPSC-CMs 

To understand the mechanisms underlying the APD-prolongation and reduction in arrhythmia-like events caused by vernakalant, the effects of vernakalant on different ion channel currents were assessed.

**Figure 4:**
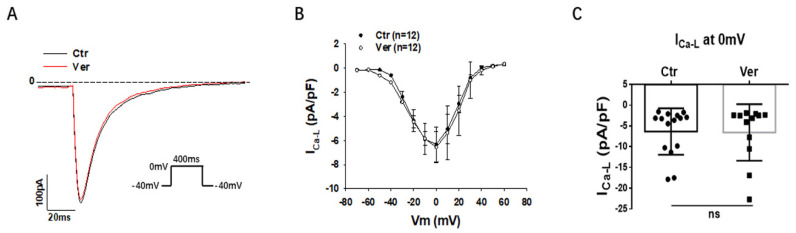
Vernakalant had no effect on L-type calcium channel currents. The L-type Ca channel currents (I_Ca-L_) were evoked by the protocol indicated in A. (**A**) The representative traces of I_Ca-L_. (**B**) Current-voltage relationship (I-V) curves of I_Ca-L_ in absence (Ctr) and presence of vernakalant (Ver, 10 µM). (**C**) Mean values of I_Ca-L_ at 0 mV in absence (Ctr) and presence of vernakalant (Ver, 10 µM). Data are shown as mean ± SEM from 15 cells. ns implies “not significant” determined by paired *t*-test.

**Figure 5:**
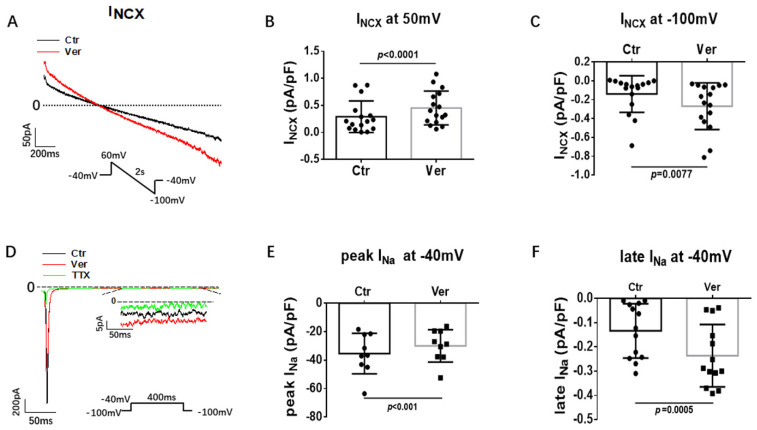
Vernakalant enhanced Na/Ca exchanger and late Na channel currents. The Na/Ca exchanger currents (I_NCX_) were evoked by the protocol indicated in A. I_NCX_ was analyzed as NiCl_2_ (5 mM) -sensitive currents. Peak and late Na channel currents (late I_Na_) were evoked by the protocol indicated in D and late I_Na_ was measured at 300 ms after initiation of the depolarization pulse. TTX (30 µM) -sensitive currents were analyzed as late I_Na_. (**A**) Representative traces of I_NCX_ in absence (Ctr) and presence of vernakalant (Ver, 10 µM). (**B**,**C**) Mean values of I_NCX_ at 60 mV and−100 mV in absence (Ctr) and presence of vernakalant (Ver, 10 µM). (**D**) Representative traces of peak and late I_Na_ in absence (Ctr) and presence of vernakalant (Ver, 10 µM). (**E**) Mean values of peak I_Na_ at −40 mV in absence (Ctr) and presence of vernakalant (Ver, 10 µM). (**F**) Mean values of late I_Na_ at −40 mV in absence (Ctr) and presence of vernakalant (Ver, 10 µM). Data are shown as mean ± SEM. *p* values were determined by paired *t*-test.

First, different inward currents were assessed. In SQTS1-hiPSC-CMs, the L-type calcium channel current (I_Ca-L_) was not significantly changed by vernakalant (Figure 4A–C). In hiPSC-CMs from the healthy donor, vernakalant significantly inhibited I_Ca-L_ (Figure S2). Moreover, the Na/Ca exchanger current (I_NCX_) was measured with NiCl_2_ (5 mM), an I_NCX_ blocker. Vernakalant enhanced I_NCX,_ at both positive and negative potentials in SQTS1-hiPSC-CMs (Figure 5A–C). In hiPSC-CMs from the healthy donor, vernakalant enhanced I_NCX_ as well (Figure S3).

Peak sodium current with a low concentration of extracellular Na concentration (20 mM) was measured. Late sodium channel current (late I_Na_) was measured with a high concentration of extracellular Na concentration (140 mM) and TTX-sensitive currents were analyzed at 300 ms after the initiation of depolarizing pulse. Under this condition, 10 µM vernakalant significantly suppressed peak I_Na_ and enhanced the late I_Na_ (Figure 5D–F). In hiPSC-CMs from the healthy donor, vernakalant exerted similar peak I_Na_ suppressing and late I_Na_ enhancing effects (Figure S4).

**Figure 6:**
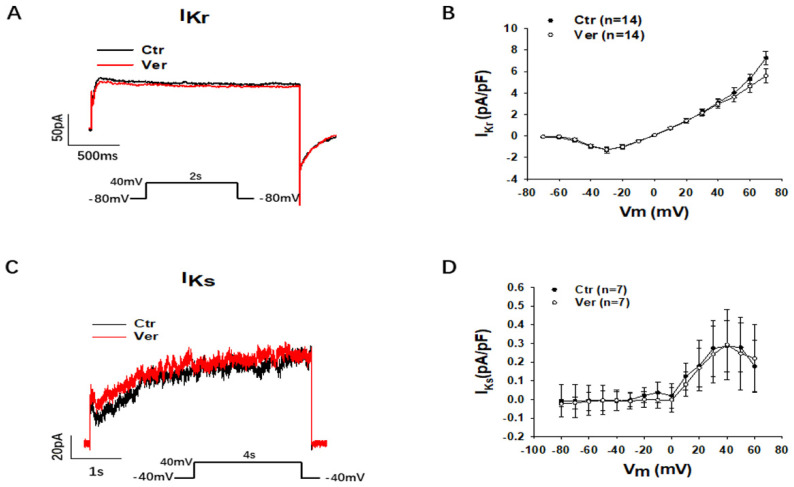
Vernakalant failed to affect I_Kr_ and I_Ks_. The currents (I_Kr_ and I_Ks_) were evoked by the protocol indicated in A and C. I_Kr_ was measured as Cs^+^ currents. I_Ks_ was analyzed as Chromalol-293B (10 µM)-sensitive currents. (**A**) Representative traces of I_Kr_ in absence (Ctr) and presence of vernakalant (Ver, 30 µM). (**B**) I-V curves of I_Kr_ in absence (Ctr) and presence of vernakalant (Ver, 30 µM). (**C**) Representative traces of I_Ks_ in absence (Ctr) and presence of vernakalant (Ver, 30 µM). (**D**) I-V curves of I_Ks_ in absence (Ctr) and presence of vernakalant (Ver, 30 µM). Data are shown as mean ± SEM.

**Figure 7:**
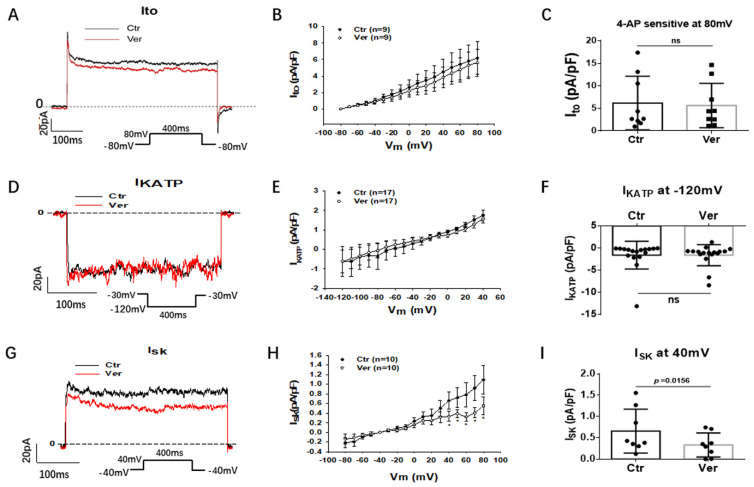
Vernakalant suppressed I_SK_ but not I_to_ and I_KATP_. The currents (I_to_, I_KATP_ and I_SK_) were evoked by the protocol indicated in A, D and G. I_KATP_ was measured as glibenclamide (10 µM) -sensitive currents. I_to_ was analyzed as 4-AP (5 mM) -sensitive currents. I_SK_ was analyzed as apamin (100 nM) -sensitive currents. (**A**) Representative traces of I_to_ at +80 mV in absence (Ctr) and presence of 30 µM vernakalant (Ver) in hiPSC-CMs from SQTS patient. (**B**) I-V curves of I_to_ in absence (Ctr) and presence of vernakalant (Ver) in hiPSC-CMs from SQTS patient. (**C**) Mean values of I_to_ at +80 mV in absence (Ctr) and presence of vernakalant (Ver) in hiPSC-CMs from SQTS patient. (**D**) Representative traces of I_KATP_ in absence (Ctr) and presence of vernakalant (Ver, 30 µM) at −120 mV. (**E**) I-V curves of I_KATP_ in absence (Ctr) and presence of vernakalant (Ver, 30 µM). (**F**) Mean values of I_KATP_ at −120 mV in absence (Ctr) and presence of vernakalant (Ver, 30 µM). (**G**) Representative traces of I_SK_ at +40 mV in absence (Ctr) and presence of vernakalant (Ver, 10 µM). (**H**) I-V curves of I_SK_ in absence (Ctr) and presence of vernakalant (Ver, 10 µM). (**I**) Mean values of I_SK_ at +40 mV in absence (Ctr) and presence of vernakalant (Ver, 10 µM). Data are shown as mean ± SEM. *p* values were determined by paired *t*-test, ns, not significant.

Then, we assessed different outward currents, including the rapidly activating delayed rectifier potassium channel current (I_Kr_) (KCNH2 or hERG channel current), the slowly activating delayed rectifier potassium channel current (I_Ks_)_,_ the transient outward current (I_to_), the ATP-sensitive K channel current (I_KATP_) and the small conductance Ca^2+^-activated K channel current (I_SK_). Vernakalant at the highest concentration (30 µM) in this study showed no significant effects on I_Kr_, I_Ks_, I_to_ and I_KATP_ but reduced I_SK_ already at 10 µM (Figure 6 and Figure 7). In hiPSC-CMs from the healthy donor, vernakalant inhibited significantly I_Kr_, I_to_ and I_SK_ at 10 µM (Figures S5–S7).

## 4. Discussion

In this study, for the first time, we tested the effects of vernakalant on APs and the occurrence of arrhythmia-like events, including DAD-like and EAD-like events in hiPSC-CMs from a patient with SQTS type 1. We observed that (1) vernakalant prolonged APD; (2) vernakalant reduced DAD-like and EAD-like events induced by CCh+Epi; (3) Vernakalant enhanced I_NCX_ and late I_Na_ and reduced I_SK_. 

Vernakalant was developed as an atrial-selective, Na and K channel-blocking drug, for the treatment of atrial fibrillation [18,19,20]. It terminates AF by prolonging the action potential duration and the effective refractory period. The APD-prolonging and anti-arrhythmic effects of vernakalant may be based on its Na and K channel-blocking effects. Its Na channel-blocking effect is voltage- and frequency-dependent, with larger blocking at lower resting membrane potential and higher frequencies [20,21]. It is well known that atrial cardiomyocytes have lower resting membrane potential than ventricular cardiomyocytes (−70 mV versus −80 mV) [22] and, thus, are more sensitive to vernakalant. On the other hand, vernakalant has been demonstrated to inhibit the potassium currents (I_Kur_, I_KAch_, and I_to_), which exist predominantly in atrial cardiomyocytes [21,23,24]. Therefore, vernakalant has been used as an atrial-selective drug for treating the recent-onset atrial fibrillation [21,25]. AF can also happen in patients with SQTS [26,27] and, hence, vernakalant can be helpful for some SQTS patients, especially those with AF. 

Vernakalant was used in a rabbit model of acquired SQTS [15]. In the rabbits injected with 1 µM pinacidil, which opens the ATP-sensitive K channels in the heart, the APD and QT intervals were shortened and the occurrence of arrhythmias (ventricular fibrillation) induced by programmed stimulations was enhanced [15]. Vernakalant prolonged the APD and QT interval and suppressed ventricular fibrillation [15]. However, the effect of vernakalant has not been tested in SQTS patients or SQTS cardiomyocytes. In the current study, we examined the APD-prolonging and antiarrhythmic effects of vernakalant in hiPSC-CMs from a patient with SQTS1 carrying the KCNH2 gene mutation of N588K (SQTS1-hiPSC-CMs), which has been confirmed as the pathogenic mutation for SQTS1 in previous studies [2,14,28]. 

In the SQTS1-hiPSC-CMs, vernakalant significantly changed APs, mainly the V_max_, APD50 and APD90. The reduction in V_max_ is consistent with its Na channel-blocking effect because V_max_ in cardiomyocytes is mainly determined by peak sodium channel currents. However, how the APD was prolonged by vernakalant in SQTS1-hiPSC-CMs needs to be clarified. 

Vernakalant has been shown to block I_Kur_, I_KAch_ and I_to_ [29], but those currents have been found predominantly in atrial myocytes. Vernakalant was also shown to block I_Kr_ in cells without hERG gene mutation [20]. This effect was confirmed in our hiPSC-CMs from a healthy donor. Since the mutation in SQTS1-hiPSC-CMs can change the sensitivity of the cell to a drug, we used the ventricular-like SQTS1-hiPSC-CMs with the KCNH2 gene mutation (N588K) for the study. The previously reported data are not enough for understanding the APD-prolonging effect of vernakalant in SQTS1-hiPSC-CMs. Thus, further ionic mechanisms underlying the APD-prolongation in SQTS1-hiPSC-CMs were investigated in this study. Because changes of either inward or outward currents may change the APD of cardiomyocytes, effects of vernakalant on different inward and outward currents were examined. 

The I_Kr_-blocking effect should be profitable for treatment of SQTS1 that results from gain-of-function in the hERG channel. Surprisingly, vernakalant failed to suppress I_Kr_ in the SQTS1-hiPSC-CMs. This indicates that the mutation of N588K in SQTS1-hiPSC-CMs caused the hERG channel to be resistant to vernakalant, in addition to other class III antiarrhythmic drugs, such as amiodarone and E-4031 [12]. I_Ks_, I_to_ and I_KATP_ are also important outward currents in cardiomyocytes. Vernakalant showed no effect on any of them in SQTS1-hiPSC-CMs, although it could inhibit them in healthy hiPSC-CMs. All these data are not in agreement with the APD-prolongation induced by vernakalant SQTS1-hiPSC-CMs.

I_SK_, another outward K channel current, can also influence APD [30,31]. We observed that vernakalant, indeed, reduced I_SK_ in our SQTS1-hiPSC-CMs and healthy hiPSC-CMs, indicative of contribution to the APD-prolongation in the presence of vernakalant. SK channels are expressed at a higher level in atrial than ventricular cardiomyocytes [31], which may also help explain the stronger effects of vernakalant in atrial than in ventricular myocytes. 

Vernakalant was shown to inhibit I_Ca-L_ with IC50 of 84 µM in the right atrial trabeculae and cardiomyocytes from patients in sinus rhythm (SR) and chronic AF [20,29,32]. In this study, we also observed a reduction in I_Ca-L_ by vernakalant in healthy hiPSC-CMs but not in SQTS1-hiPSC-CMs. This may be due to the mutation in SQTS1-hiPSC-CMs. However, how the mutation in the hERG channel altered the drug effect on I_Ca-L_ is difficult to understand and needs to be explored in future studies.

The inward current I_NCX_ and late I_Na_ were also enhanced by vernakalant, which has not been reported before. This effect may also be involved in the APD-prolongation by vernakalant.

The APD-prolonging effect may counteract the arrhythmogenic effect of APD shortening. Therefore, we tested the possible effects of vernakalant on arrhythmia-like events in SQTS1-hiPSC-CMs. Indeed, it reduced occurrence of arrhythmia-like events (EAD-or DAD-like events) induced by CCh+Epi, suggesting vernakalant might be a candidate for treating SQTS1. The vernakalant-induced reduction in arrhythmia-like events may result from its APD-prolonging and Na channel-blocking effects.

The hiPSC-CMs possess spontaneously beating property. On one hand, because the mature cardiomyocytes possess no automaticity under physiological conditions, the spontaneous beating may reflect the immaturity in cell structure and functions, including some intracellular signal transduction and Ca^2+^ homeostasis. On the other hand, the spontaneous beating may show arrhythmia-like events and, hence, can provide a possibility to investigate drug effects on arrhythmia-like events at the cell level. It was shown that calcium transients in spontaneously beating hiPSC-CMs could be used to monitor arrhythmia-like events in cells from patients with Brugada syndrome [33]. In our study, vernakalant could reduce arrhythmia-like events monitored by calcium transients in beating cells and enhance I_NCX_ or late I_Na_, leading to APD prolongation. An increased I_NCX_ or late I_Na_ can elevate intracellular Na^+^ concentration. The increase in intracellular Na^+^ concentration can increase the intracellular Ca^2+^ via the reverse-mode function of Na/Ca exchanger [34,35] and, in turn, may facilitate Ca^2+^-associated arrhythmias. However, vernakalant did not elevate intracellular Ca^2+^ concentration (the baseline and amplitude of calcium transients were not changed). From data in this study, it is unclear how vernakalant reduced arrhythmia-like events induced by Epi+CCh. Probably, vernakalant can exert effects on some intracellular calcium handling proteins or their associated signaling. 

Though human adult cardiomyocytes could provide more relevant information than hiPSC-CMs, the availability of adult cardiomyocytes from patients is limited. On the other hand, AP can be influenced by many ion channel currents. For screening effects of vernakalant on different channel currents and APD, hiPSC-CM showed advantages, considering its unlimited availability and its characterization, similar to human adult cardiomyocytes, as well as its ability to recapitulate the phenotypic features of cardiac disorders, although the hiPSC-CM has its own limitations. 

## 5. Conclusions

The results demonstrated that vernakalant led to APD prolongation and a reduction in arrhythmia-like events, probably through its effects on I_NCX_, late I_Na_ and I_SK,_ besides Na channel blocking. Vernakalant may have beneficial effects in SQTS1 patients and may be a candidate drug for SQTS1 treatment.

## 6. Study Limitations

We recruited only one SQTS1 patient for this study. Differences among individuals cannot be ruled out. However, the study may be relevant for personalized precision medicine. 

Immaturity is another limitation of hiPSC-CMs to be considered. The possibility that responses of mature cardiomyocytes in SQTS patients to vernakalant are different from that of the SQTS1-hiPSC-CMs cannot be excluded. The real benefit of vernakalant still needs to be clarified in SQTS patients. 

## Data Availability

The data that support the findings of this study are available within the manuscript and Supplementary Material.

## Supplementary Materials

The following supporting information can be downloaded at: https://www.mdpi.com/article/10.3390/jcdd9040112/s1, Figure S1: Effects of vernakalant on action potentials in hiPSC-CMs from healthy donor. Figure S2: Effect of vernakalant on L-type calcium channel currents in hiPSC-CMs from healthy donor. Figure S3: Vernakalant enhanced Na/Ca exchanger and late Na channel currents in hiPSC-CMs from healthy donor. Figure S4: Effect of vernakalant on peak and late Na channel currents in hiPSC-CMs from healthy donor. Figure S5: Effect of vernakalant on IKr in hiPSC-CMs from healthy donor. Figure S6: Effect of vernakalant on ISK in hiPSC-CMs from healthy donor. Figure S7: Effect of vernakalant on Ito in hiPSC-CMs from healthy donor.
