# Supplementary material for: Antiarrhythmic Effects of Vernakalant in Human-Induced Pluripotent Stem Cell-Derived Cardiomyocytes from a Patient with Short QT Syndrome Type 1
Source: J Cardiovasc Dev Dis. 2022 Apr 9;9(4):112. doi: 10.3390/jcdd9040112 (PMC9032933; doi:10.3390/jcdd9040112)
Supplement: Supplementary file 1 [file jcdd-09-00112-s001.zip › jcdd-1615644-supplementary.pdf]

Supplemental information

Antiarrhythmic effects of vernakalant in human-induced pluripotent stem cell-derived cardiomyocytes from a patient with short QT syndrome type 1

Running title: Vernakalant effects in SQTS1-hiPSC-CMs

Qiang Xu[13], Xuemei Huang[2], Zenghui Meng[3], Yingrui Li[3], Rujia Zhong[3], Xin Li[3], Lukas Cyganek[5,6], Ibrahim El-Battrawy[3,4], Ibrahim Akin[3,4], Xiaobo Zhou[3,4,2*], Huan Lan[2*]

[1] School of Basic Medical Science, Southwest Medical University, Luzhou, China.

[2]Key Laboratory of Medical Electrophysiology, Ministry of Education & Medical Electrophysiological Key Laboratory of Sichuan Province, Collaborative Innovation Center for Prevention of Cardiovascular Diseases, Institute of Cardiovascular Research, Southwest Medical University, Luzhou, China;

[3]First Department of Medicine, Faculty of Medicine, University Medical Centre Mannheim (UMM), University of Heidelberg, Mannheim, Germany;

[4]DZHK (German Center for Cardiovascular Research), Partner Site, Heidelberg-Mannheim, Germany;

[5]Stem Cell Unit, Clinic for Cardiology and Pneumology, University Medical Center Göttingen, Göttingen, Germany;

[6]DZHK (German Center for Cardiovascular Research), Partner Site, Göttingen, Germany;

*Correspondence: Huan Lan, E-mail: lanhuan@swmu.edu.cn; Xiaobo Zhou, E-mail: Xiaobo.zhou@medma.uni-heidelberg.de;

Xiaobo Zhou and Huan Lan share senior authorship.

Figure legends

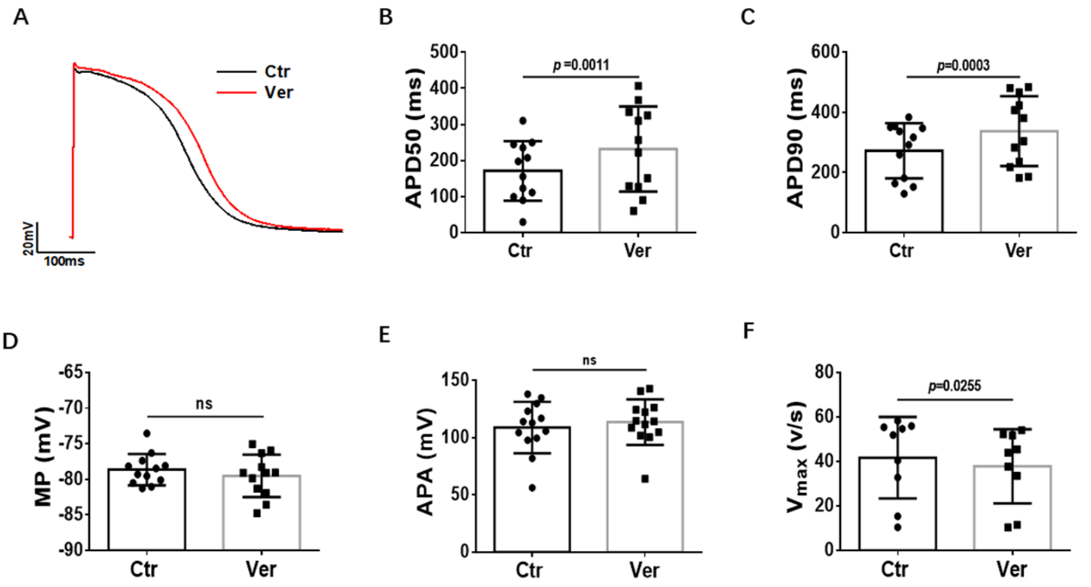

Figure S1. Effects of vernakalant on action potentials in hiPSC-CMs from healthy donor. (A) Representative action potential traces in absence (Ctr) and presence of 10 µM vernakalant (Ver). (B) Averaged values of action potential duration at 50% repolarization (APD50). (C) Averaged values of action potential duration at 90% repolarization (APD90). (D) Averaged values of resting potential (RP). (E) Averaged values of action potential amplitude (APA). (F) Averaged values of maximal depolarization velocity (V_{max}). All the action potentials were recorded at 1 Hz. Shown are mean ± SEM, n represents number of cells. The statistical significance was examined paired t-test.

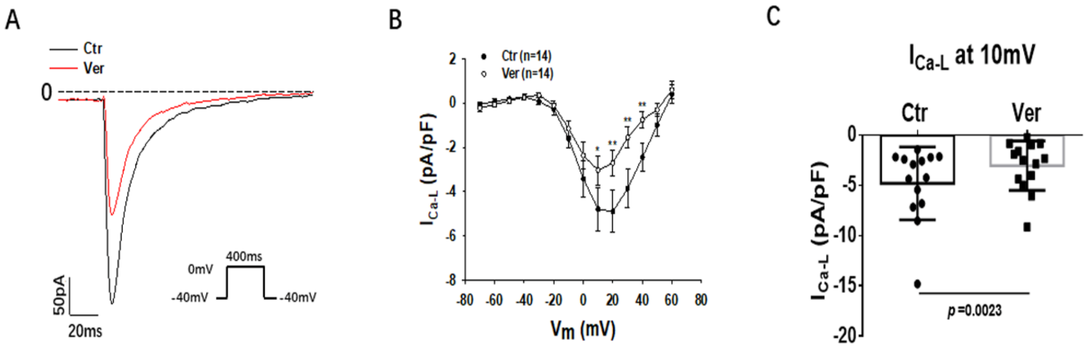

Figure S2. Effect of vernakalant on L-type calcium channel currents in hiPSC-CMs from healthy donor. The L-type Ca channel currents ($I_{Ca\text{-}L}$) were evoked by the protocol indicated in A. (A) The representative traces of $I_{Ca\text{-}L}$ in absence (Ctr) and presence of 10 µM vernakalant (Ver). (B) Current-voltage relationship (I-V) curves of $I_{Ca\text{-}L}$ in absence (Ctr) and presence of vernakalant (Ver). (C) Mean values of $I_{Ca\text{-}L}$ at 10 mV in absence (Ctr) and presence of vernakalant (Ver). Shown are mean ± SEM.

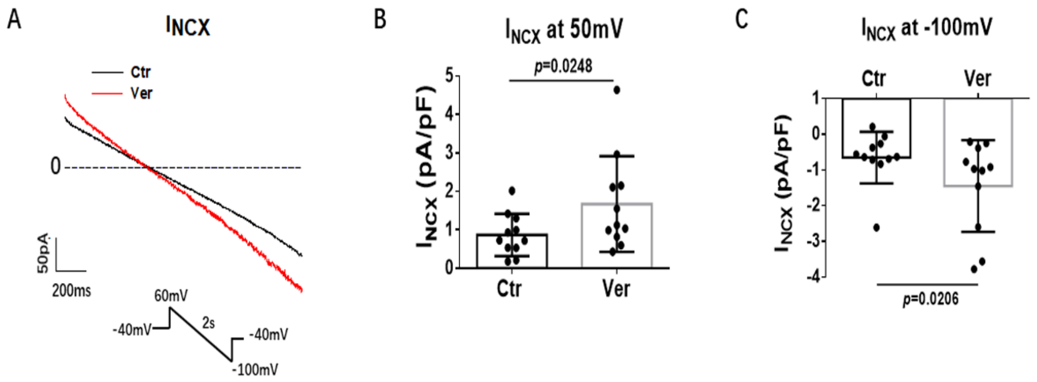

Figure S3. Vernakalant enhanced Na/Ca exchanger and late Na channel currents in hiPSC-CMs from healthy donor. The Na/Ca exchanger currents (I_{NCX}) were evoked by the protocol indicated in A. I_{NCX} was analyzed as $NiCl_2$ (5 mM)

sensitive currents. (A) Representative traces of I_{NCX} in absence (Ctr) and presence of 10 µM vernakalant (Ver). (B, C) Mean values of I_{NCX} at 60 mV and -100 mV in absence (Ctr) and presence of vernakalant (Ver).

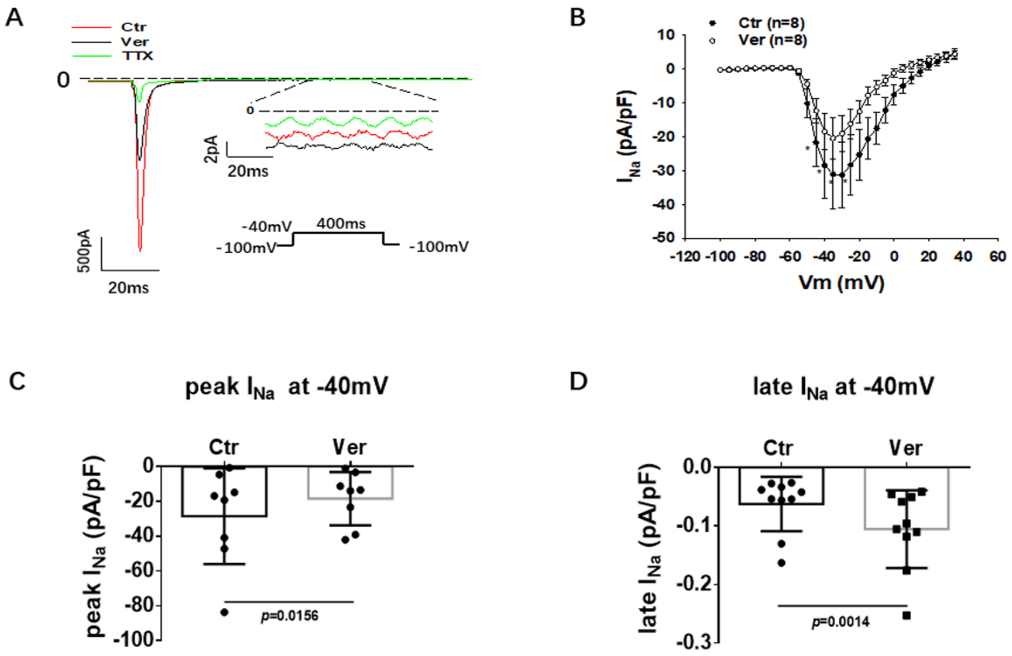

Figure S4. Effect of vernakalant on peak and late Na channel currents in hiPSC-CMs from healthy donor. Peak and late Na channel currents (INa) were evoked by the protocol indicated in A. Late INa was measured at 300 ms after initiation of the depolarization pulse. TTX (30 µM) sensitive currents were analyzed as late I_{Na}. (A) Representative traces of peak and late I_{Na} in absence (Ctr) and presence of 10 µM vernakalant (Ver). (B) I-V curves of peak INa in absence and presence of vernakalant (Ver). (C) Mean values of peak I_{Na} at -40 mV in absence (Ctr) and presence of vernakalant (Ver). (D) Mean values of late I_{Na} at -40 mV in absence (Ctr) and presence of vernakalant (Ver).

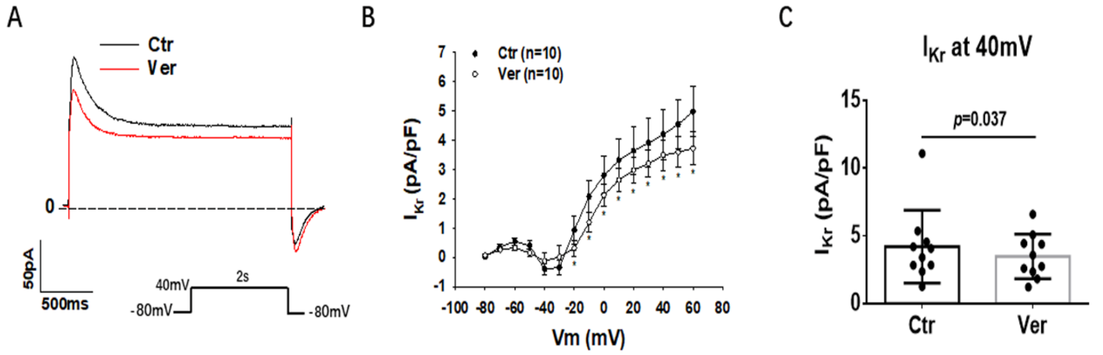

Figure S5. Effect of vernakalant on I_{Kr} in hiPSC-CMs from healthy donor. The I_{Kr} currents were evoked by the protocol indicated in A. I_{Kr} was measured as Cs^+ currents. I_{Ks} was analyzed as Chromalol-293B (10 µM) sensitive currents. (A) Representative traces of I_{Kr} in absence (Ctr) and presence of 10 µM vernakalant (Ver). (B) I-V curves of I_{Kr} in absence (Ctr) and presence of vernakalant (Ver). (C) Mean values of I_{Kr} at 40 mV in absence (Ctr) and presence of vernakalant (Ver).

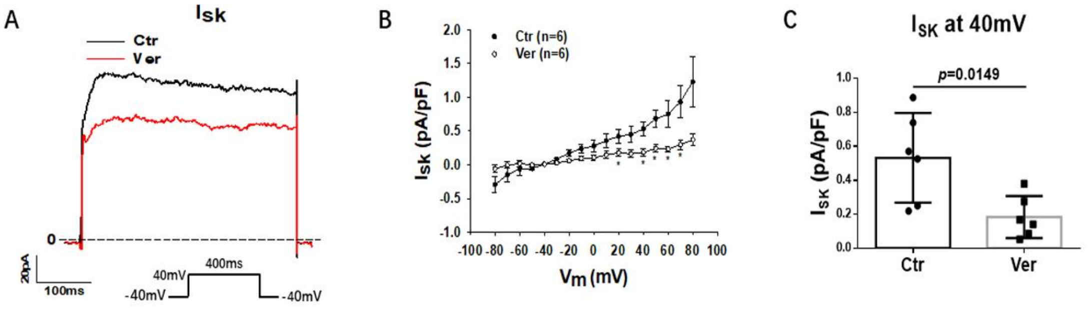

Figure S6. Effect of vernakalant on I_{SK} in hiPSC-CMs from healthy donor. The I_{SK} currents were evoked by the protocol indicated in A. I_{SK} was analyzed as apamin (100 nM) sensitive currents. (A) Representative traces of I_{SK} at +40 mV in absence (Ctr) and presence of 10 µM vernakalant (Ver). (B) I-V curves of I_{SK}

in absence (Ctr) and presence of vernakalant (Ver). (C) Mean values of I_{SK} at +40 mV in absence (Ctr) and presence of vernakalant (Ver).

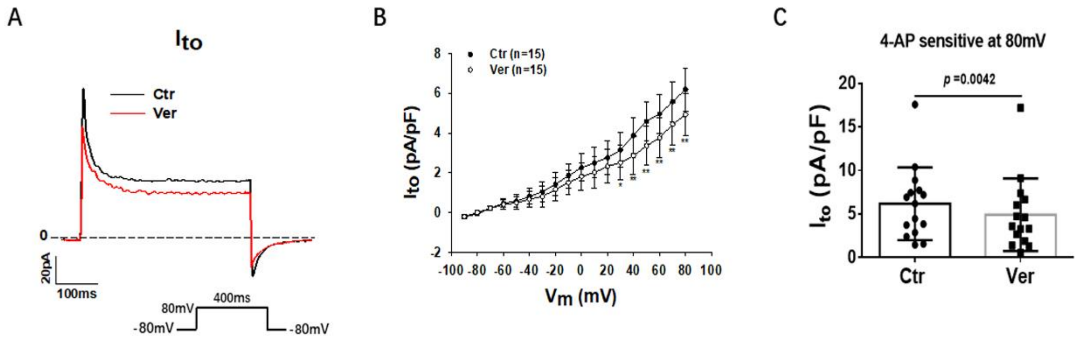

Figure S7. Effect of vernakalant on I_{to} in hiPSC-CMs from healthy donor. The I_{to} currents were evoked by the protocol indicated in A. I_{to} was analyzed as 4-AP (5 mM) sensitive currents. (A) Representative traces of I_{to} at +80 mV in absence (Ctr) and presence of 10 µM vernakalant (Ver) in hiPSC-CMs from healthy donor. (B) I-V curves of I_{to} in absence (Ctr) and presence of vernakalant (Ver) in hiPSC-CMs from healthy donor. (C) Mean values of I_{to} at +80 mV in absence (Ctr) and presence of vernakalant (Ver) in hiPSC-CMs from healthy donor.
